# Is social support associated with hypertension control among Ghanaian migrants in Europe and non-migrants in Ghana? The RODAM study

**DOI:** 10.1007/s11739-019-02075-7

**Published:** 2019-03-26

**Authors:** Gertrude Nsorma Nyaaba, Karien Stronks, Karlijn Meeks, Erik Beune, Ellis Owusu-Dabo, Juliet Addo, Ama de-Graft Aikins, Frank Mockenhaupt, Silver Bahendeka, Kerstin Klipstein-Grobusch, Liam Smeeth, Charles Agyemang

**Affiliations:** 1 Department of Public Health, Amsterdam University Medical Centres, Amsterdam Public Health (APH) Research Institute, University of Amsterdam, Meibergdreef 9, 1105 AZ Amsterdam, The Netherlands; 2 Barcelona Institute for Global Health (ISGlobal), University of Barcelona, Barcelona, Spain; 3 School of Public Health, Kwame Nkrumah University of Science and Technology, Kumasi, Ghana; 4 Department of Non-communicable Disease Epidemiology, London School of Hygiene and Tropical Medicine, London, UK; 5 Regional Institute for Population Studies, University of Ghana, Legon, Ghana; 6 Charité—Universitaetsmedizin Berlin and Institute of Tropical Medicine and International Health, Berlin, Germany; 7 MKPGMS—Uganda Martyrs University, Kampala, Uganda; 8 Julius Global Health, Julius Centre for Health Sciences and Primary Care, University Medical Centre Utrecht, Utrecht, The Netherlands; 9 Division of Epidemiology and Biostatistics, School of Public Health, Faculty of Health Sciences, University of the Witwatersrand, Johannesburg, South Africa

**Keywords:** Hypertension control, Social support, Sub-Saharan Africa, Migrant health, Ethnic minority groups, Ghana, Europe

## Abstract

Hypertension (HTN) control is crucial in preventing HTN-related complications such as stroke and coronary heart disease. Yet, HTN control remains suboptimal particularly among sub-Saharan African (SSA) populations partly due to poor self-management. Self-management of HTN is influenced by social support, but the evidence on the role of social support on HTN control particularly among SSA populations is limited. This study assessed the association between multiple proxies for social support and HTN control among Ghanaians resident in Ghana and Europe. The Research on Obesity and Diabetes among African Migrants (RODAM) study participants with HTN and who self-reported HTN (*n* = 1327) were included in this analysis. Logistic regression was used to assess the association between proxies of social support and HTN control (SBP < 140 mmHg and DBP < 90 mmHg) with adjustments for age and socioeconomic status (SES). Among Ghanaian males in both Europe and Ghana, cohabiting with more than two persons was associated with increased odds of having HTN controlled. Male hypertensive patients cohabiting with ≥ 5 persons had the highest odds of having HTN controlled after adjustment for age and SES (OR 0.30; 95% CI 0.16–0.57; 0.60; 0.34–1.04, respectively). This association was not observed among females. Relationship status, frequency of religious activity attendance and satisfaction with social support did not show any significant association with HTN control. Our study shows that cohabitation is significantly associated with HTN control but in males only. The other proxies for social support appeared not to be associated with HTN control. Involving persons living with Ghanaian men with HTN in the treatment process may help to improve adherence to HTN treatment. Further research is needed to explore in-depth, how these social support proxies could contribute to improved HTN control among SSA populations.

## Introduction

Evidence shows a high prevalence of hypertension (HTN) but suboptimal control rates for HTN among Sub-Saharan African (SSA) origin groups in Europe [1–6]. Recent studies on the Research on Obesity and Diabetes among African Migrants (RODAM) study show a 56.8% and a 34.4% HTN prevalence among Ghanaian migrants living in Europe and Ghanaians in Ghana with suboptimal HTN control rates of 20% and 52.5% among Ghanaian migrant men in Europe and non-migrant rural Ghanaian men, respectively [5, 7]. Adequate blood pressure (BP) control (i.e. systolic and diastolic BP (SBP/DBP) less than 140/90 mmHg [8]) considerably lessens the rate of cardiovascular events such as stroke, renal insufficiency, coronary heart disease, peripheral vascular disease, congestive heart failure and premature deaths [3, 9, 10].

The reasons for the poor control of HTN among SSA origin groups remain unclear although untreated severe HTN, differences in the prescription practices, fear of side effects and poor patient self-management (patient’s daily activities surrounding adherence to medication and behavioural recommendations) are thought to be potential underlying factors [5, 11]. While it is generally accepted that behavioural factors and low socioeconomic status (SES) contribute to individual’s risks for HTN [12, 13], the extent to which social support mechanisms could influence HTN control has been relatively unexplored especially among SSA origin populations. Evidence shows that social support contributes significantly to improving health outcomes [14–16]. A review on the influence of social support on chronic illness self-management shows that social support has a protective effect on self-management particularly in supporting dietary changes [17]. The bulk of evidence on social support has been on the characteristics of family support such as perceived family support [17, 18], family solidity, articulateness and struggles [17, 19], which enable adherence to treatment for chronic conditions. Data on other social support indicators such as cohabitation, relationships, patient satisfaction with social support and social networks such as religious attendance are lacking. This suggests the need for further research on the role of other key indicators of social support on chronic illness self-management.

Within African societies where members live together with shared responsibilities and possessions, studies show that during times of illness, members play a “brokerage function” between patients and healers and are regarded as the *“*therapy managing group” [20]. A recent study on HTN self-management in Nigeria found religion and marital status as significant determinants of social support, which contributed to adherence to HTN treatment [16]. Another study on community perceptions of HTN in rural Ghana found that social support could contribute to improvement in adherence to treatment particularly for adherence to dietary regimes and HTN medication [21]. Evidence indicates that social support is one of the main determinants of adherence to HTN treatment among SSA migrants living in high income countries (HICs) [22]. While these studies [16, 21, 22] have provided evidence on the role of social support to adherence to HTN treatment, the specific indicators of social support, which could influence HTN control among SSA migrants, have not yet been clearly elucidated. Identifying specific indicators of social support, which could enhance patient self-management of HTN is key to identifying pathways for implementing targeted interventions to improve HTN control among these populations.

The RODAM study provides an opportunity to assess the association between social support and HTN control as it used a highly standardised approach to collect data among homogenous SSA populations living in different locations in Europe and in rural and urban Ghana [23]. Using data from the RODAM study, we assessed the association between social support and HTN control among Ghanaians resident in Europe and Ghana and whether the associations varied in the different contexts. We hypothesised that living with more people, been in a relationship, regular religious activity attendance and high satisfaction with social support received would positively influence HTN control among SSA migrants and non-migrants.

## Methods

### Data source

Data from the RODAM study, which was carried out from 2012 to 2015, were used for this analysis. The study received ethical approval in all participating countries and included Ghanaians aged 25–70 years residing in three European countries (The Netherlands, Germany and UK) and Ghanaians living in rural and urban Ghana; and all participants gave written consent before data collection. The rationale, conceptual framework, methodology, response rates and other details have been published elsewhere [5, 23].

### Description of measurements

The RODAM study used a structured questionnaire to collect information on sociodemographic, medical history, treatment and lifestyle, and psychosocial stress. Validated devices were used to conduct physical examination according to standardised operational procedures across all study sites. A validated semi-automated device (the Microlife WatchBP home Widnau, Switzerland) with appropriate cuffs was used to measure BP three times in a sitting position after at least, a 5-min rest. HTN was defined as SBP ≥ 140 mmHg or DBP ≥ 90 mmHg and or being on antihypertensive medication. HTN awareness was defined as the proportion of individuals with HTN who reported HTN prior to BP measurements by the study team [5]. HTN treatment was defined as the proportion of persons with HTN who had been prescribed antihypertensive medication for high BP management, while HTN was defined as controlled if a participant was on antihypertensive medication with BP < 140/90 mmHg [5].

### Study design and measurements

**Fig. 1:**
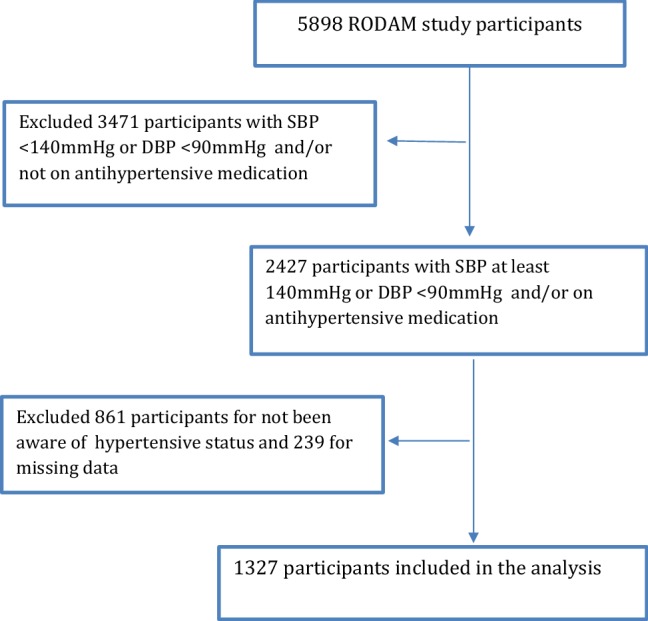
Sample selection

We selected Ghanaian participants from the RODAM study with HTN. Because social support requires a degree of patient awareness of HTN status, we excluded participants who were unaware of their HTN status. We therefore conducted our analysis on 1327 participants who were hypertensive and were aware of their HTN status (Fig. 1).

A person’s social network often evolves social contacts [24] around the place of abode, work, relationship and religion. Four proxies for social support were used in this analysis: number of cohabitants, relationship status, religious attendance frequency and satisfaction with social support received. Participants recorded the number of persons living with them in their household (cohabitants) and responded to their relationship status. Based on the distribution of the relationship status responses, participant responses were re-categorised into a binary variable (yes/no) where “yes” represents married/registered partnership and cohabiting, while “no” represents unmarried, divorced or separated and widow/widower. In addition, frequency of religious activity attendance was categorised into two groups (once a week or more and less than once a week) based on the distribution of participant responses. Furthermore, because we were interested in patient satisfaction with social support received, the Social Support Scale for Satisfaction with supportive transactions (SSQS) was used to measure five items (warmth, friendliness, willingness to lend a friendly ear, are not problem-oriented and affection of people in the participant’s environment) on a 1–4 point Likert scale. Answer categories were ordinal and included much less than I like (1), less than I like (2), just as much as I like (3) and more than I like (4). Higher scores indicated more satisfaction with social support received. Based on the distribution of the responses, we categorised responses into low, medium and high levels of satisfaction with social support received.

### Data analysis

Characteristics of study participants were expressed as means and standard deviation (SD) for continuous variables and percentages for categorical variables. Logistic regression was used to assess the association between the four proxies for social support and HTN control. Logistic regression models were adjusted for age, sex and SES. Level of education, occupation, income or a composite of these factors are most commonly used to determine SES [25]. For this study, SES refers to the level of education and employment status of a participant as they are key socially derived economic factors that influence the status held by participants within their societies. Based on the distribution of the employment status responses, participants were categorised into employed and unemployed where employed referred to participants who were actively engaged in income generating activities. We found significant statistical interactions between sex and some of the proxies for social support in relation to HTN control and therefore stratified the analyses by sex. All analyses were performed using IBM SPSS Statistics 24.

## Results

### Sociodemographic characteristics

Table 1 presents a detailed description of study participants’ sociodemographic characteristics. The mean age of all participants was 53.2 (± 9.0), and the average number of years that migrants had lived in Europe was 21.2 (± 9.5). HTN was uncontrolled in 68% of all participants most of whom had lower vocational or secondary education (35.8%). While 26.9% were non-migrant Ghanaians, 73.1% were migrant Ghanaians living in Europe. Among men, HTN was uncontrolled in 41.6% of study participants. Of all the participants, 36% were fully employed, 73.7% were using antihypertensive medication and males accounted for 36.3% of the total population. 3.8% of migrants reported current smoking compared with 0.8% of non-migrants. 61.7% of non-migrants and 29.8% of migrants reported being on a special diet because of HTN. More than half of non-migrant participants (55.2%) and less than half of migrants (40.6%) were married or were in registered partnerships. The average number of cohabitants was 5.2 (± 2.6) for non-migrants and 3.3 (± 1.6) for migrants. The majority of the participants attended religious activities at least once a week (85.4%), and 58.7% of patients expressed a medium satisfaction with social support received.

**Table 1:** Characteristics of participants selected for analysis (*n* = 1327)

	All			Ghana			Europe		
	Total	Hypertension controlled	Hypertension uncontrolled	Total	Hypertension controlled	Hypertension uncontrolled	Total	Hypertension controlled	Hypertension uncontrolled
	*n* = 1327	*n* = 424	*n* = 903	357 (26.9)	105 (29.4)	252 (70.6)	970 (73.1)	319 (32.9)	651 (67.1)
**Demographics**									
Age, years (SD)	53.1 (9.0)	53.9 (8.3)	52.7 (9.3)	55.2 (10.5)	55.8 (8.9)	54.9 (11.1)	52.3 (8.3)	53.3 (7.9)	51.9 (8.4)
**Site**									
Ghana	357 (26.9)	105 (24.8)	252(27.9)	357 (100)	105 (29.4)	252 (70.6)	NA	NA	NA
Rural Ghana	235 (65.8)	35 (33.3)	87 (34.5)	235 (65.8)	35 (33.3)	87 (34.5)	NA	NA	NA
Urban Ghana	122 (34.2)	70 (66.7)	165 (65.5)	122 (34.2)	70 (66.7)	165 (65.5)	NA	NA	NA
Europe	970 (73.1)	319(75.2)	651(72.1)	NA	NA	NA	970 (100)	319 (32.9)	651 (67.1)
Amsterdam	468 (48.2)	168 (52.7)	300 (46.1)	NA	NA	NA	468 (48.2)	168 (52.7)	300 (46.1)
Berlin	194 (20.0)	47 (14.7)	147 (22.5)	NA	NA	NA	194 (20.0)	47 (14.7)	147 (22.5)
London	308 (31.8)	104 (32.6)	204 (31.3)	NA	NA	NA	308 (31.8)	104 (32.6)	204 (31.3)
**Sex**									
Male	482 (36.3)	106 (25.0)	376 (41.6)	88 (24.6)	21 (20.0)	67 (26.6)	394 (40.6)	85 (26.6)	309 (47.5)
Female	845 (63.7)	318 (37.6)	527 (62.3)	269 (75.4)	84 (31.2)	185 (68.8)	576 (59.4)	234 (40.6%	342 (59.4)
**Length of stay in Europe (years)**									
Mean (SD)	NA	NA	NA	NA	NA	NA	21.2 (9.5)	21.4 (9.3)	21.0 (9.5)
1–18	NA	NA	NA	NA	NA	NA	338 (36.7)	111 (34.8)	227 (34.9)
19–25	NA	NA	NA	NA	NA	NA	319 (34.6)	113 (35.4)	206 (31.6)
26–64	NA	NA	NA	NA	NA	NA	264 (28.7)	83 (26.0)	181 (27.8)
**Education completed**									
Elementary or less	436 (33.3)	133 (31.8)	303 (34.0)	200(56.0)	52 (49.5)	148 (58.7)	236 (24.8)	81 (25.9)	155 (24.3)
Lower vocation or lower secondary	481 (36.7)	162 (38.8)	319 (35.8)	115 (32.2)	43 (41.0)	72 (28.6)	366 (38.4)	119 (38.0)	247 (38.7)
Intermediate vocational or intermediate secondary	269 (20.6)	93 (22.2)	176 (19.8)	31 (3.1)	8 (7.6)	23 (9.1)	238 (25.0)	85 (27.2)	153 (23.9)
Higher vocation or university	123 (9.4)	30 (7.2)	93 (10.4)	11 (3.1)	2 (1.9)	9 (3.6)	112 (11.8)	28 (8.9)	84 (13.1)
**Employment status**									
Employed full-time	470 (36.0)	147 (35.1)	323 (36.4)	73 (20.4)	22 (21.0)	51 (20.2)	397 (41.8)	125 (39.8)	272 (42.8)
Employed part-time	388 (29.7)	119 (28.4)	269 (30.3)	204 (57.1)	55 (52.4)	149 (59.1)	184 (19.3)	64 (20.4)	120 (18.9)
Retired	88 (6.7)	33 (7.9)	55 (6.2)	22 (6.2)	7 (6.7)	15 (6.0)	66 (6.9)	26 (8.3)	40 (6.2)
Unemployed	75 (5.7)	20 (4.8)	55 (6.2)	4 (1.1)	2 (1.9)	2 (0.8)	71 (7.5)	18 (5.7)	53 (8.3)
Unable to work	131 (10.0)	47 (11.2)	84 (9.5)	49 (13.7)	19 (18.1)	30 (11.9)	82 (8.6)	28 (8.9)	54 (8.5)
On social benefits	118 (9.0)	43 (10.3)	75 (8.5)	2 (0.6)	–	2 (0.8)	116 (12.2)	43 (13.6)	73 (11.5)
Full-time homemaker	30 (2.3)	8 (1.9)	22 (2.5)	3 (0.8)	–	3 (1.2)	27 (2.8)	8 (2.5)	19 (2.9)
Student	7 (0.5)	2 (0.5)	5 (0.6)	–	–	–	7 (0.7)	2 (0.6)	5 (0.8)
**Hypertension management characteristics**									
Use of antihypertensive medication	977 (73.6)	424 (100)	553 (61.2)	209 (58.5)	105 (100)	104 (41.3)	768 (79.2)	319 (100)	449 (69.0)
Smoking, % Yes	40 (3.0)	8 (1.9)	32 (3.6)	3 (0.8)	–	3 (1.2)	37 (3.8)	8 (2.5)	29 (4.5)
Special diet for HTN, % Yes	508 (40.4)	182 (45.3)	326 (38.2)	219 (61.7)	75 (72.1)	144 (57.4)	289 (29.8)	107 (35.9)	182 (30.2)
Alcohol, % No	818 (61.6)	270 (63.7)	548 (60.7)	251 (70.3)	77 (73.3)	174 (69.0)	567 (58.5)	193 (60.5)	374 (57.5)
*Physical activity*									
Low	420 (37.3)	132 (31.1)	288 (27.8)	146 (41.0)	45 (42.9)	101 (40.2)	274 (35.5)	87 (33.3)	187 (36.7)
Moderate	214 (19.0)	69 (16.3)	145 (19.1)	53 (14.9)	14 (13.3)	39 (15.5)	161 (20.9)	55 (21.1)	106 (20.8)
High	493 (37.2)	165 (38.9)	328 (42.1)	157 (44.0)	46 (43.8)	111 (44.2)	336 (43.6)	119 (45.6)	217 (42.5)
*Types of cohabitants*									
Living with partner	631 (60.5)	201 (59.0)	430 (60.8)	204 (60.0)	62 (62.6)	142 (58.9)	427 (60.7)	139 (58.6)	288 (61.8)
Living with children 3 and younger	184 (19.7)	61 (19.5)	123 (18.3)	77 (22.8)	23 (23.7)	54 (22.5)	107 (16.5)	38 (17.6)	69 (16.0)
Living with children 4 years and older	781 (75.6)	253 (76.2)	528 (75.3)	309 (91.4)	90 (90.9)	219 (91.6)	472 (67.9)	163 (70.0)	309 (66.9)
Living with other adults	186 (18.6)	53 (16.8)	133 (19.4)	54 (15.9)	19 (19.4)	35 (14.5)	132 (19.9)	34 (15.7)	98 (22.0)
**Social support-related characteristics**									
*Number of cohabitants*									
Mean (SD)	3.9 (2.1)	3.9 (2.1)	3.9 (2.1)	5.2 (2.6)	5.3 (2.6)	5.2 (2.5)	3.3 (1..6)	3.4 (1.5)	3.3 (1.7)
1–2 cohabitants	369 (30.3)	111 (28.7)	258 (31.0)	56 (15.7)	15 (14.3)	41 (16.3)	313 (36.3)	96 (34.0)	217 (37.4)
3–4 cohabitants	443 (36.3)	147 (38.0)	296 (35.6)	90 (25.2)	23 (21.9)	67 (26.6)	353 (41.0)	124 (44.0)	229 (39.5)
5 or more cohabitants	407 (33.4)	129 (33.3)	278 (33.4)	211 (59.1)	67 (63.8)	144 (57.1)	196 (22.7)	62 (22.0)	134 (23.1)
*Frequency of religious attendance*									
Once a week or more	910 (85.4)	302(86.3)	608 (84.9)	261 (95.3)	74 (96.1)	187 (94.9)	649 (81.9)	228 (83.5)	421 (81.1)
Once every 2 weeks	51 (4.8)	18 (5.1)	33 (4.6)	3 (1.1)	2 (2.6)	1 (0.5)	48 (6.1)	16 (5.9)	32 (6.2)
Once a month	41( 3.8)	8 (2.3)	33 (4.6)	3 (1.1)	–	3 (1.5)	38 (4.8)	8 (2.9)	30 (5.8)
Less than once a month	36 (3.4)	13 (3.7)	23 (3.2)	4 (1.5)	–	4 (2.0)	32 (4.0)	13 (4.8)	19 (3.7)
Never	28 (2.6)	9 (2.6)	19 (2.7)	3 (0.8)	1 (1.3)	2 (1.0)	25 (3.2)	8 (2.9)	17 (3.3)
*Relationship status*									
Married/registered partnership	580 (44.6)	168 (40.8)	412 (46.3)	197 (55.2)	57 (54.3)	140 (55.6)	383 (40.6)	111 (36.2)	272 (42.7)
Cohabiting	133 (10.2)	48(11.7)	85 (9.6)	25 (7.0)	9 (8.6)	16 (6.3)	108 (11.4)	39 (12.7)	69 (10.8)
Unmarried	154 (11.8)	49 (11.9)	105 (11.8)	6 (1.7)	1 (1.0)	5 (2.0)	148 (15.7)	48 (15.6)	100 (15.7)
Divorced or separated	331(25.4)	110 (26.7)	221(24.9)	51 (14.3	15 (14.3)	36 (14.3)	280 (29.7)	95 (30.9)	185 (29.0)
Widow/widower	103 (7.8)	37 (9.0)	66 (7.4)	78 (21.8)	23 (21.9	55 (21.8)	25 (2.6)	14 (4.6)	11 (1.7)
*Satisfaction with social support received*									
Low	329 (25.7)	107 (26.0)	222 (25.6)	86 (24.4)	30 (28.8)	56 (22.5)	243 (26.2)	77 (25.1)	166 (26.8)
Medium	751 (58.7)	240 (58.4)	511 (58.9)	232 (65.7)	64 (61.5)	168 (67.5)	519 (56.0)	176 (57.3)	343 (55.4)
High	199 (15.6)	64 (15.6)	135 (15.6)	35 (9.9)	10 (9.6)	25 (10.0)	164 (17.7)	54 (17.6)	110 (17.8)

Data are *n* (%)

*SD* standard deviation, *NA* not applicable

No cases = –

### Social support and hypertension control

The association between number of cohabitants as a proxy for social support and HTN control is presented in Table 2a. Among all males, cohabiting with two persons or less or with 3–4 persons was associated with lower odds of having controlled HTN compared to those cohabiting with five or more persons after adjustment for age and SES [odds ratio (OR) 0.30; 95% CI 0.16–0.57; 0.60; 0.34–1.04, respectively]. The odds were similar among male migrants in Europe and male non-migrants in Ghana when the analysis was stratified by site. No significant association was observed among migrant and non-migrant females even after adjusting for age and SES (OR 1.26; 95% CI 0.86–1.85 for cohabiting with 3–4 persons; and 1.27; 0.89–1.81 for cohabiting with five persons or more, respectively). The association between cohabiting and HTN in females did not vary by geographical locations (Europe and Ghana).

**Table 2:** The association between number of cohabitants, relationship status and hypertension control stratified by sex

Model	Sex	Location	2a. Cohabitation			2b. Relationship status	
			1–2 cohabitants OR (95% CI)	3–4 cohabitants OR (95% CI)	5 and more cohabitants OR (95% CI)	Yes OR (95% CI)	No OR (95% CI)
Model 1: Crude + age	Male	All	0.31(0.16–0.59)	0.60 (0.35–1.04)	1.00 (Ref)	1.00 (Ref)	0.99 (0.62–1.58)
		Europe	0.34 (0.16–0.70)	0.67 (0.35–1.29)			1.06 (0.64–1.76)
		Ghana	–	0.21 (0.04–1.03)			0.46 (0.05–3.98)
Model 2: Model 1 + Educational status and Employment		All	0.30 (0.16–0.57)	0.60 (0.34–1.04)	1.00 (Ref)	1.00 (Ref)	0.94 (0.58–1.51)
		Europe	0.32 (0.15–0.68)	0.66 (0.34–1.29)			1.05 (0.62–1.78)
		Ghana	–	0.28 (0.5–1.48)			0.40 (0.04–3. 3.65)
Model 1: Crude + age	Female	All	1.28(0.88–1.86)	1.29 (0.91–1.82)	1.00 (Ref)	1.00 (Ref)	0.97 (0.73–1.29)
		Europe	1.13(0.68–1.87)	1.28 (0.79–2.06)			1.00 (0.71–1.41)
		Ghana	0.99(0.49–2.05)	0.88 (0.46–1.64)			0.82 (0.47–1.43)
Model 2: Model 1 + Educational status and Employment		All	1.26(0.86–1.85)	1.27 (0.89–1.81)	1.00 (Ref)	1.00 (Ref)	0.98 (0.72–1.32)
		Europe	1.12 (0.67–1.88)	1.13 (0.75–1.71)			1.05 (0.72–1.52)
		Ghana	0.99 (0.48–2.07)	0.87 (0.46–1.65)			0.82 (0.46–1.44)

– No cases

There was no association between relationship status and HTN control in both sexes and across sites as shown in Table 2b. Among all males, the OR was 0.94 (95% CI 0.58–1.51), while in all females the OR was 0.98 (95% CI 0.72–1.32).

Table 3a, b shows the sex stratified associations between frequency of attendance of religious activities and satisfaction with social support received and HTN control. After adjusting for age, there was no significant association between HTN control and frequency of attendance of religious activities in all males or all females and across sites. In addition, satisfaction with social support received was not associated with HTN control in both sexes. In both Europe and Ghana, neither medium nor high satisfaction with social support received was associated with HTN control in both sexes. The models that were additionally adjusted for SES showed similar results.

**Table 3:** The association between hypertension control and frequency of attendance of religious activities and perceived social support received stratified by sex

Model	Sex	Location	3a. Religious activities attendance		3b. Satisfaction with social support received		
			Once a week or more OR (95% CI)	Less than once a week OR (95% CI)	Low OR (95% CI)	Medium OR (95% CI)	High OR (95% CI)
Model 1: Crude + age	Male	All	1.00 (Ref)	1.10 (0.58–2.07)	1.00 (Ref)	0.79 (0.48–1.28)	0.58 (0.28–1.21)
		Europe		1.09 (0.57–2.10)		0.82 (0.48–1.40)	0.57 (0.26–1.27)
		Ghana		–		0.67 (0.21–2.17)	0.63 (0.09–4.11)
Model 2: Model 1 + educational status and employment		All	1.00 (Ref)	1.06 (0.55–2.03)	1.00 (Ref)	0.79 (0.49–1.31)	0.59 (0.28–1.23)
		Europe		1.06 (0.54–2.08)		0.82 (0.47–1.43)	0.57 (0.25–1.18)
		Ghana		–		0.59 (0.17–2.15)	0.34 (0.04–2.56)
Model 1: Crude + age	Female	All	1.00 (Ref)	1.06 (0.61–1.83)	1.00 (Ref)	0.99 (0.70–1.39)	1.13 (0.71–1.78)
		Europe		1.03 (0.57–1.87)		1.22 (0.80–1.86)	1.25 (0.73–2.12)
		Ghana		0.79 (0.15–4.08)		0.67 (0.37–1.23)	0.71 (0.27–1.90)
Model 2: Model 1 + educational status and employment		All	1.00 (Ref)	0.98 (0.56–1.742	1.00 (Ref)	0.99 (0.71–1.42)	1.15 (0.72–1.82)
		Europe		0.94 (0.52–1.73)		1.19 (0.78–1.83)	1.27 (0.74–2.17)
		Ghana		0.76 (0.15–4.02)		0.71 (0.38–1.33)	0.68 (0.25–1.86)

– No cases

## Discussion

### Key findings

Our findings show that living with more than two other persons increases the odds of having HTN controlled among migrant and non-migrant Ghanaian men but not among migrant and non-migrant Ghanaian women. There was no significant association between the other social support proxies (relationship status, frequency of religious activity attendance and satisfaction with social support received) and HTN control in both sexes.

### Discussion of the key findings

Migrant and non-migrant Ghanaian males were more likely to have HTN controlled if they lived with more than two people, but this association was not observed among female migrants and non-migrants. Our findings are consistent with the findings of a study conducted in England, where living alone was negatively associated with HTN control among English males but not among English females [26]. On average, non-migrants live with five other persons in a household, which is in line with the documented average Ghanaian national household size of 4.0 [27]. Migrants living in Europe on the other hand, live with an average of three other persons in a household, which is slightly higher than the 2.3 persons per European Union (EU) household size in 2016 reported by Eurostat. Within African societies, cohabitants potentially comprise members of the same lineage including relatives such as parents, siblings, spouses, children, uncles and aunts who form the extended family. Evidence shows that treatment and health care evolve around kinship (common lineage) systems within African societies with relatives brokering and facilitating health care for members during times of ill health [20, 28]. Male patients might therefore feel more comfortable discussing their illness and or health problems with their cohabitants than their female counterparts. Among all study participants, over 60% lived with their partners, over 70% lived with children 4 years and above, and over 18% lived with other adults who could provide both direct and indirect social support in facilitating patients adherence to medication through reminders to take medication and adherence to dietary recommendations. For instance, nearly half of the participants with controlled HTN were on special HTN diet, which may be facilitated by living with partner or older child or other adults. Indeed, a study found that African American men recognise the role of having supportive family relations, particularly females, in controlling HTN as they support them to integrate treatment recommendations into their daily lives [29]. Female members, however, may not receive such support during their HTN treatment possibly, because traditionally, females are primarily caregivers during periods of ill health in African societies. Another possible explanation could be that females may have broader social networks and interactions, which provide them with alternative social support mechanisms beyond family or other extended kin living with them, which could potentially explain why cohabitation was not significantly associated with HTN control among females. Existing evidence shows that females have wider sources of emotional support and may not necessarily consider their partners as their closest support [30].

Contrary to our hypothesis, we did not observe a significant association between relationship status and HTN control for both sexes and in migrants and non-migrants. Several studies have shown the association between marital status and HTN control particularly for males [24, 26, 31], and thus, we anticipated similar associations in our study population. It is plausible that given the extended nature of the African family system, social support maybe offered directly and/or indirectly by other relations or adult children and not necessarily from a partner. As such, even among migrants, while being in a relationship was not associated with HTN control, living with more than two other persons was associated with HTN control in men.

Evidence shows that the SSA migrant populations in Europe are close knitted and organised around social structures evolving around community and faith-based or religious organisations, which provide social support systems for their members [32]. Studies have shown that social networks have an influence on cardiovascular health outcomes [33–36]. As such, we hypothesised that hypertensive patients who attended religious actives at least weekly, would have increased odds of having their HTN controlled compared with those that did not attend religious activities weekly. Contrary to our hypothesis, we did not find any association between regular attendance at religious activities and HTN control. While the lack of association observed in our study is similar to the results of an earlier study conducted in southern Africa [37], other studies have shown a negative association between HTN control and social networks among males in Spain and African American males in North Carolina, USA [24, 38]. It may well be that such gatherings at religious activities may not present the right context or environment for patients to discuss their HTN status. While participation in religious activities provides opportunities for social interactions, which are particularly common within the Ghanaian culture, they may not necessarily build sufficient trust networks for hypertensive patients to discuss their health condition, concerns or share experiences. This is particularly more evident among males who may be apprehensive of disclosing or discussing their HTN status given the connotation between antihypertensive medication and sexual weakness, which has been shown to be a barrier to medication adherence among males in this population [11]. In addition, the fear of stigma due to HTN has been found to be a key reason for non-disclosure of hypertension status to community members [11] and this could potentially explain the lack of association between this kind of social support and HTN control as patients may not have disclosed their HTN status and hence religious members may not even be aware of a person’s status. A recent qualitative study on community perceptions of HTN found that non-disclosure of HTN status was a key barrier to members ability to support members with HTN [21]. The frequency of religious attendance may not fully capture the social processes and interactions surrounding regular attendance at religious activities. For instance, some religious groups such as the Pentecostal Council of Churches (PCC) in the Netherlands have been shown to have more health-centred activities [32] and an individual’s personal involvement may influence the kind of social support religious groups or members provide [39].

The association between satisfaction with social support and BP control has been reported in other studies [24, 40], but in this study, the odds were similar for high and medium patient satisfaction when compared with low patient satisfaction with social support received. However, given the sociocultural and communal relationships predominant within African communities, the perception of what constitutes social support might differ between our study population and other populations. For instance, younger family members and children may directly facilitate adherence to medication by reminding patients to take medication while females may facilitate adherence to dietary recommendations by ensuring that meals are salt free and serving meals together with HTN medication for male HTN members. These activities may not be perceived as forms of social support by patients within African societies because they are expected and considered societal norms and duties of family members during times of ill health as they play the “brokerage function” of the “therapy managing group” identified by Janzen et al. In addition, sociocultural norms and practices within Ghanaian communities frown on smoking habits, which potential explains the low (3%) prevalence of current smokers recorded in this study and thus facilitating adherence to smoking recommendations.

Our results show that of the four proxies for social support, only cohabitation was associated with HTN control among men while none of the social support proxies was associated with HTN control among females. The lack of association between social support and HTN control among females has also been reported among older women in Spain [24]; and a study conducted in England showed that low perceived social support was associated with low HTN control rates [26]. While the key social indicators that could contribute to improving HTN control among men are unravelling, the underlying mechanisms which could potentially improve HTN control among females, particularly among female African migrants, remain unclear. Perhaps, it is reasonable to assume that given the health carer role that females, in particular African females play within the family and community system, they do not perceive themselves as having similar access to socially supportive resources or spousal support as their male counterparts. For instance, if a woman is diagnosed with HTN, adherence to dietary changes such as reduction of salt content in meals may be hindered by the inability of relatives to accept such dietary changes because of relatives’ poor recognition of their own risk to developing HTN [21, 41]. Given that only 37.6% of all female participants had HTN controlled and the health caretaker roles that females assume in this context, if efforts are not made to address HTN control rates among females, the rates of uncontrolled HTN among African females may supersede the rates of uncontrolled HTN among males in coming years. Additional studies are therefore needed to identify and explore the specific social support mechanisms that could contribute to improved adherence and HTN control among SSA females.

### Limitations

The main limitation to this study was that the definition of HTN was based on three blood pressure (BP) measurements on a single occasion as in most epidemiological studies. However, it is unlikely that this differs between the groups under study and thus this limitation would not affect the associations observed in this study. Moreover, despite a standardised research protocol, because of differences in registration systems in the various sites, recruitment had to be modified to suit the settings, which could account for lower sample size recorded in some sites. In addition, we were unable to further stratify the analysis by religious groups due to small sample size for HTN control. Furthermore, we lack data on other factors relating to cohabitation such as the numbers and gender of children living with hypertensive study participants, which may influence HTN control. Lastly, the quantitative nature of the questionnaire hinders extrapolations about the association between these social support proxies and HTN control as the underlying concepts of these proxies may not be fully captured, which could account for the associations or lack of associations observed.

## Conclusion

Our study findings show that living with more than two people is positively associated with HTN control among migrant and non-migrant Ghanaian males. It provides evidence of the role of cohabitation as a key determinant for social support, which enhances HTN management among male patients. Further research is needed to explore in-depth, how SSA patients conceptualise social support in order to identify modifiable determinants and the specific social support mechanisms that could contribute to adherence to HTN treatment and HTN control among SSA populations.
